# A defective structural zipper in photoreceptors causes inherited blindness

**DOI:** 10.1371/journal.pbio.3001672

**Published:** 2022-06-17

**Authors:** Siebren Faber, Ronald Roepman

**Affiliations:** Department of Human Genetics and Radboud Institute for Molecular Life Sciences, Radboud University Medical Center, Nijmegen, the Netherlands

## Abstract

This Primer explores a PLOS Biology study which uses ultrastructure expansion microscopy to study the inner scaffold of the photoreceptor connecting cilium, the location of multiple proteins implicated in inherited forms of progressive sight loss such as retinitis pigmentosa and Leber congenital amaurosis.

## Teaser

Being able to see the beauty of this world is a wonderful thing unfortunately unavailable to people with inherited blindness. In this issue of *PLOS Biology*, Mercey and colleagues present optimized expansion microscopy for retinal tissue, which represents a huge step forward in our ability to study these blinding conditions.

Vision is one of the most valuable senses of the human body, as it allows us to see the magnitude and beauty of this world. Seeing begins when rays of light enter the eye via the cornea, are refracted by the lens, and projected to the back of the eye (https://webvision.med.utah.edu). The projection at the back of the eye can be compared to the way an image is created by a digital film camera. Instead of a digital sensor, the back of the eye is decorated with several layers of interconnected neurons, called the retina (https://webvision.med.utah.edu). The outermost layer of the retina, last reached by the light, consists of millions of light-sensitive cells, known as the photoreceptors [1]. The photoreceptors consist of morphological and functional distinct cellular compartments, including a synaptic terminal, an inner segment, an outer segment, and a connecting cilium. The main function of the photoreceptors is carried out in the outer segments, where phototransduction takes place, a strictly organized light-converting cascade involving many proteins. All of these proteins are synthesized in the inner segments and transported to the outer segments through the connecting cilium, making this an important bridge between these compartments.

The importance of this bridge becomes apparent when genes encoding for proteins localizing to the connecting cilium are mutated, often leading to retinitis pigmentosa (RP) and Leber congenital amaurosis (LCA), the most frequent and most severe form of inherited blindness, respectively [2]. Despite the known localization of these RP- and LCA-associated proteins at the level of the connecting cilium, their exact role and localization within this compartment remain to be determined. The main reason of the poor characterization of the connecting cilium proteins in previous studies is the limited resolution that can be reached by conventional fluorescence microscopy methods.

Mercey and colleagues now overcome this limitation by, for the first time, applying ultrastructure expansion microscopy (U-ExM) on retina tissue [3]. By implementing this technique, they are able to physically expand the specimen by an average factor of 4.2 times, while preserving the interconnected neuronal cell layers of the retina. Since the expansion is water based, the final expanded gel consists predominantly of water, making the specimen transparent and less susceptible to optical aberration. This makes it ideally suited for imaging using the user-friendly conventional fluorescence microscopes, with higher image acquisition and processing speeds compared to classic super-resolution techniques, while obtaining similar or even better resolution [4].

**Fig 1:**
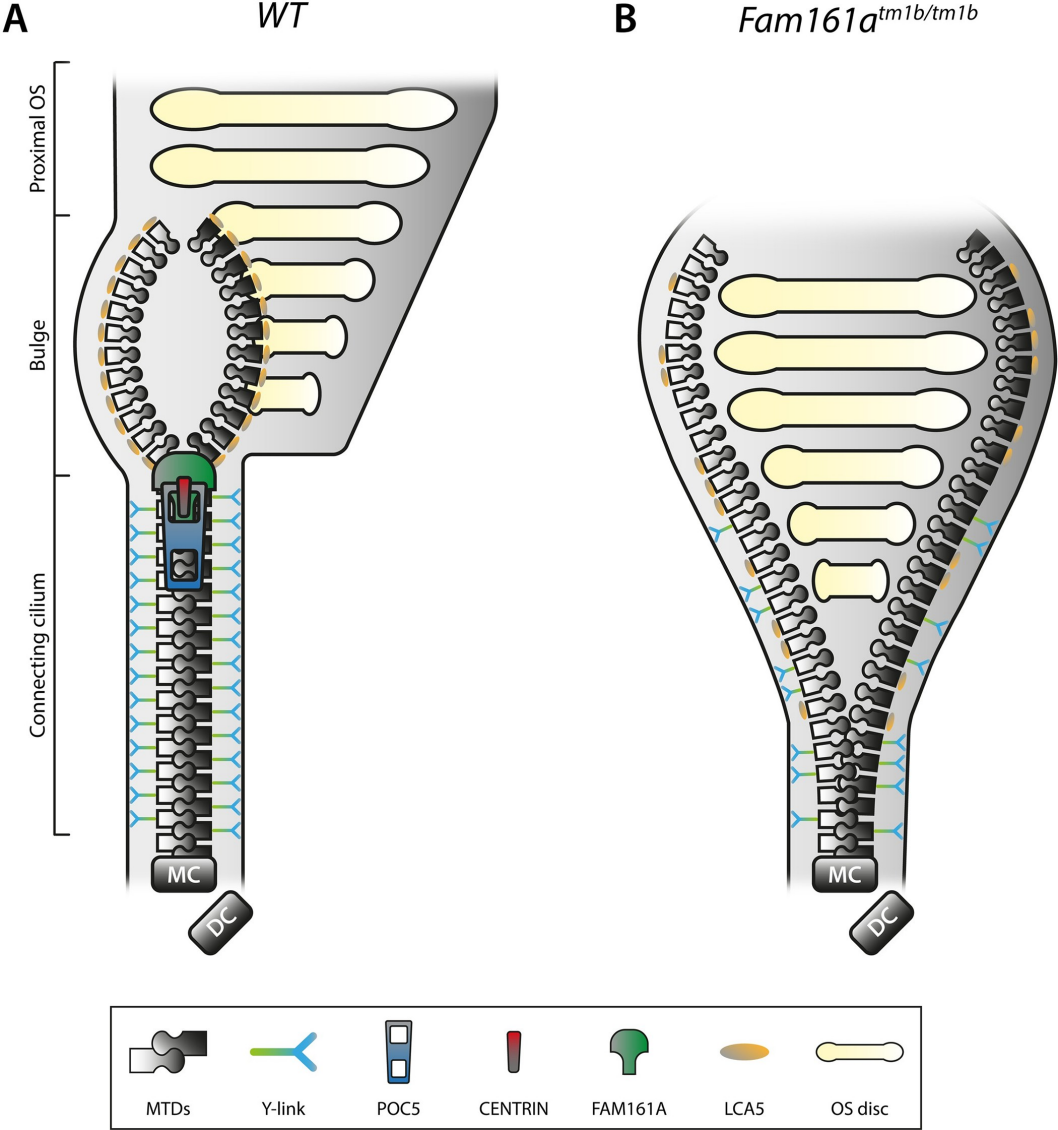
The CC inner scaffold acts as structural zipper providing coherence between the MTDs. **(A)** Schematic representation of a part of a WT rod photoreceptor consisting of the CC, the bulge region, and the proximal OS, including its membranous stacked discs. The MTDs are built up from the MC, accompanied by the DC. Cohesion of the MTDs in the CC is maintained by the inner scaffold proteins POC5, CENTRIN, and FAM161A, located at the inner wall of the MTDs, comparable with a closed zipper. Please note that these proteins are found all along the CC, in addition to the MC and DC (not shown in this diagram). MTDs in the CC are connected to the membrane by Y-links, associated with CEP290 and SPATA7 localization. LCA5 localizes to the bulge region, where MTDs are more dispersed due to the absence of the inner scaffold and Y-links. **(B)** Deficiency of FAM161A causes loss of the entire zip head (the CC inner scaffold) as also POC5 and Centrin are absent, leading to spreading of the MTDs. This spreading, visualized by an open zipper, eventually causes a collapse of the OS structure. Protein localization at the Y-links level is secondarily affected when FAM161A is depleted, as seen by more dispersed CEP290 localization. Furthermore, FAM161A deficiency results in disorganization of the bulge region, obvious from LCA5 localizing more proximal to the MC. Altogether, the CC inner scaffold forms a structural foundation securing proper disc formation and OS integrity. DC, daughter centriole; CC, connecting cilium; MC, mother centriole; MTD, microtubule doublet; OS, outer segment; WT, wild-type.

The physical expansion leads to a spatial distancing of the molecules, making them more accessible for antibodies, which could improve the antibody specificity and binding capacity, eventually resulting in better distinguishable protein localization patterns. Indeed, Mercey and colleagues were able to show distinct localization of several proteins within the tidily organized connecting cilium ultrastructure, thereby revealing the existence and the composition of the connecting cilium inner scaffold [3]. This inner scaffold provides coherence between the 9 microtubule doublets, comparable with a structural zipper (Fig 1A). Consistent with their earlier work on centrioles [5], they now identified CENTRIN, POC5, and FAM161A along the full length of the connecting cilium at the level of the inner scaffold, inside the microtubule doublets [3]. Although the connecting cilium localization of CENTRIN was shown before at high resolution by electron microscopy (EM) [6], the relative localization of CENTRIN to the microtubule doublets could not be determined, due to the limitation of the colorless single labeling that was used.

Taking advantage of the possibility for double labeling with tubulin and CEP290 on the expanded retinas, Mercey and colleagues could show that CEP290 localizes along the full length of the connecting cilium outside of the microtubule doublets with a previously unidentified [7], 9-fold symmetry consistent with the Y-links region [3]. They also found a highly similar localization pattern of SPATA7, a presumed functional interactor of CEP290 [8].

By identifying the connecting cilium inner scaffold as structural zipper, Mercey and colleagues identified a distinct region, directly above the connecting cilium, which they called the “bulge region.” They found that this region was lacking both the inner scaffold and the Y-links, resulting in more dispersed microtubule doublets [3]. Lebercilin, encoded by the *LCA5* gene, specifically localizes to this bulge region, in accordance with previous EM analysis [9], with a 9-fold symmetry in the extension of CEP290. Mutations in LCA5 cause LCA [9], highlighting the importance of this region for photoreceptor viability.

To validate the idea of the connecting cilium functioning as structural zipper, Mercey and colleagues assessed the photoreceptor development of FAM161A mutant mice from early postnatal to adult stage [3]. Indeed, they found that the coherence between microtubule doublets at the level of the connecting cilium was lost due to the absence of the connecting cilium inner scaffold/structural zipper in the mutant mice (Fig 1B). The spreading of the microtubule doublets eventually results in a collapse of the outer segment structure, obvious from the rhodopsin localization in adult FAM161A deficient mice. This phenotype strongly correlates with the phenotype observed in *SPATA7* mutant mice, as shown by cryo-electron tomography [8]. This phenotypical resemblance is remarkable, since Mercey and colleagues showed the independence between the connecting cilium inner scaffold and the Y-links, confirmed by difference in distribution of POC5 and CEP290 in both developing and degenerating photoreceptors [3].

It would be of major interest to investigate the interplay between the connecting cilium inner scaffold, the Y-links, the bulge region, and the microtubule doublets in context of various inherited blinding conditions. For this, the optimized U-ExM by Mercey and colleagues [3] can be applied on various mutant mouse models for inherited blindness. U-ExM on developing *CEP290* and *SPATA7* mutant retinas will expand previous knowledge on neonatal mouse retinas at the level of Y-links, analyzed by EM and stochastic optical reconstruction microscopy (STORM) [7,8]. Further research on developing LCA5 KO mouse retinas by U-ExM will provide crucial insights in the novel identified bulge region. Additionally, proteins like PCARE and RP1 could potentially also be linked to the bulge area by U-ExM, as they are shown to localize apical to the connecting cilium by EM and confocal microscopy, respectively [10,11].

The application of U-ExM on retinal tissue thus opens new directions in understanding the biology of photoreceptors and the etiology of inherited blindness. Similar applications to other neuronal tissues and organoid models may, in the future, undoubtedly prove to be equally valuable.

## Abbreviations

EM: electron microscopy
LCA: Leber congenital amaurosis
RP: retinitis pigmentosa
STORM: stochastic optical reconstruction microscopy
U-ExM: ultrastructure expansion microscopy
